# 

**Published:** 1873-10-15

**Authors:** 


Rock River Medical Society.—The next meeting of Rock River Medical Society (Wis.) will be held at the Rock River House, in Theresa, Saturday, Nov. 1, 1873, at 10 o'clock A.M. Subject for discussion: "The Causes, Symptoms, Pathology, and Treatment of Flexions of the Uterus." Essayist, Dr. William J. Burns. All physicians of the surrounding counties, in good standing, are cordially invited to attend.

